# Infections with *Pseudomonas aeruginosa* in patients admitted to the “Victor Babeş” Clinical Hospital of Infectious Diseases and Pneumology, Craiova

**DOI:** 10.1186/1471-2334-13-S1-P40

**Published:** 2013-12-16

**Authors:** Dan Dumitru Hurezeanu, Livia Dragonu, Carmen Canciovici, Daniela Ristea, Doina Ene, Mioara Cotulbea, Magdalena Peter, Felicia Dumitraşcu, Maria Bălan

**Affiliations:** 1 University of Medicine and Pharmacy Craiova, Romania; 2 “Victor Babeş” Clinical Hospital of Infectious Diseases and Pneumology, Craiova, Romania; 3 Emergency Clinical Hospital, Craiova, Romania

## Background

*Pseudomonas aeruginosa* has a high resistance to external environmental conditions, with a remarkable ability to adapt and numerous virulence factors, being involved in the etiology of nosocomial infections and severe infections in immunodeficient patients. The strains isolated in hospitals need supervision due to the risk of transmission and antibiotic resistance.

We pursued the epidemiological context to elaborate therapeutic recommendations on first-line antibioterapy adapted to the evolution of antibiotic resistance of *Pseudomonas* strains isolated from pathological products collected from hospitalized patients in non-ICU departments of the hospital.

## Methods

The retrospective study included 192 patients diagnosed with infections caused by *P aeruginosa* hospitalized in the “Victor Babeş” Hospital Craiova in the period 01 January 2006 – 31 December 2012. We evaluated the studied cases based on the following parameters: the risk of infection with antibiotic-resistant germs (Carmeli score), infection localization and antibiotic resistance profile of the isolated strains of *P aeruginosa*, achieved by the classical diffusimetric system.

## Results

The annual evolution of cases showed an upward trend since 2009, the age groups most affected were infants (8.3% of cases), small children (11.1%) and the elderly over 65 years (26.5%). According to the Carmeli score, patients were divided into 3 categories: 4 patients (2.1%) with community infections, 112 patients (58.4%) with community apparent infections and 76 patients (39.5%) with probable nosocomial infections. The location of infection was: respiratory (49.6%), ear (16.1%), urinary (12.8%), digestive (10.1%), overinfected wounds (2.7%), sepsis (1.6%), other sites (ocular, articular) 1%.

Antibiotic sensitivity was: colistin 99.1%, quinolone 88.7%, cefoperazone-sulbactam 88%, aminoglycosides 87.7%, meropenem 84.4%, aztreonam 83.9%, piperacillin-tazobactam 82.14% ticarcillin-clavulanate 77.3%, ceftazidime 73.8%, cefpirome 72.7%, ceftriaxone 71.6%, cefepime 60.9%, amoxicillin-clavulanate 16.1%. The comparative analysis of antibiotic sensitivity according to the Carmeli score indicates caution in the use of cephalosporins and quinolones for patients with Carmeli score 3, since these patients probably have infections with antibiotic resistant strains. The risk for strains of *P aeruginosa* to be carbapenem-resistant showed respiratory and urinary localization and wound infections. Colistin was the safest alternative treatment for *P aeruginosa* strains resistant to meropenem, becoming the most effective antibiotic in the absence of its use for a long time.

## Conclusion

The antibiotic resistance of *P aeruginosa* was determined by antibiotic selection pressure, the chemosensitivity data are useful in developing therapeutic recommendations tailored to the local situation.

